# Precise Detection of *IDH1/2* and *BRAF* Hotspot Mutations in Clinical Glioma Tissues by a Differential Calculus Analysis of High-Resolution Melting Data

**DOI:** 10.1371/journal.pone.0160489

**Published:** 2016-08-16

**Authors:** Ryusuke Hatae, Nobuhiro Hata, Koji Yoshimoto, Daisuke Kuga, Yojiro Akagi, Hideki Murata, Satoshi O. Suzuki, Masahiro Mizoguchi, Koji Iihara

**Affiliations:** 1 Department of Neurosurgery, Graduate School of Medical Sciences, Kyushu University, Fukuoka, Japan, 3-1-1 Maidashi, Higashi-ku, Fukuoka 812–8582, Japan; 2 Department of Neurosurgery, Clinical Research Institute, National Hospital Organization, Kyushu Medical Center, Fukuoka, Japan; 3 Department of Neuropathology, Graduate School of Medical Sciences, Kyushu University, Fukuoka, Japan; 4 Department of Neurosurgery, Kitakyushu Municipal Medical Center, Kitakyushu, Japan; Sapporo Ika Daigaku, JAPAN

## Abstract

High resolution melting (HRM) is a simple and rapid method for screening mutations. It offers various advantages for clinical diagnostic applications. Conventional HRM analysis often yields equivocal results, especially for surgically obtained tissues. We attempted to improve HRM analyses for more effective applications to clinical diagnostics. HRM analyses were performed for *IDH1*^*R132*^ and *IDH2*^*R172*^ mutations in 192 clinical glioma samples in duplicate and these results were compared with sequencing results. *BRAF*^*V600E*^ mutations were analyzed in 52 additional brain tumor samples. The melting profiles were used for differential calculus analyses. Negative second derivative plots revealed additional peaks derived from heteroduplexes in PCR products that contained mutations; this enabled unequivocal visual discrimination of the mutations. We further developed a numerical expression, the HRM-mutation index (MI), to quantify the heteroduplex-derived peak of the mutational curves. Using this expression, all *IDH1* mutation statuses matched those ascertained by sequencing, with the exception of three samples. These discordant results were all derived from the misinterpretation of sequencing data. The effectiveness of our approach was further validated by analyses of *IDH2*^*R172*^ and *BRAF*^*V600E*^ mutations. The present analytical method enabled an unequivocal and objective HRM analysis and is suitable for reliable mutation scanning in surgically obtained glioma tissues. This approach could facilitate molecular diagnostics in clinical environments.

## Introduction

Gliomas account for over 30% of all primary brain tumors, and the majority require adjuvant treatments, including chemotherapy and/or radiotherapy [1]. Recent advances in high-throughput sequencing have unveiled various somatic mutations in gliomas [2–4]. Among these, several “hotspot mutations” located in specific regions leading to gains-of-function related to gliomagenesis have been identified [5]. *IDH1/2* hotspot mutations have been observed in the vast majority of astrocytomas and oligodendrogliomas [6–7], and the *BRAF*^*V600E*^ mutation is frequently seen in pleomorphic xanthoastrocytomas, gangliogliomas, extra-cerebellar pilocytic astrocytoma, and a subset of diffuse gliomas [8–10]. Clinical applications of mutation analyses for both *IDH1/2* and *BRAF*^*V600E*^ are highly anticipated, not only for molecular diagnostics, but also for decision-making regarding molecular-targeted therapies.

Immunohistochemistry (IHC) and Sanger sequencing (SS) are conventionally performed to detect glioma-related mutations; however, clinical applications of these analyses are still limited. One of the primary issues that limit the clinical applications is the intrinsic heterogeneity of gliomas; highly sensitive platforms are required to detect genetic alterations [11]. Tolerance to heterogeneity is essential for the genetic analysis of glioma samples.

High resolution melting (HRM) analysis is an alternative technique developed to screen for variation in DNA sequences. The development of novel saturation dyes has enabled the creation of the HRM platform, which is based on the principle that variation in DNA sequences produces alterations in the melting characteristics of DNA amplicons [12–14]. Clinical applications of this technique are less time-consuming, simpler, and more sensitive than previous techniques [15]. Homozygous samples typically have symmetric melting transitions, whereas heterozygotes show a more complex transition owing to the presence of different homo- and hetero-duplexes, which are discriminated by differences in melting curve shapes [13]. Because *IDH1/2* and *BRAF*^*V600E*^ mutations are heterozygous, HRM is a suitable method for their detection in clinical environments [16]. However, conventional HRM platforms determine mutations using pre-equipped software that differentiates samples with respect to a normalized wild-type melting curve [4,17]. Accordingly, the subjective nature of interpretation (i.e., the lack of a distinct indicator) remains an unresolved issue in HRM analyses [18]. Conventional HRM analyses of clinical tissue samples have provided equivocal results more frequently than other platforms [18]. Unequivocal determinations are essential for the clinical diagnosis of gliomas, which are characterized by high intrinsic heterogeneity.

In the present study, a differential calculus analysis was established in order to develop an unequivocal method for mutation scanning using HRM. The effectiveness of this method was explored using *IDH1/2* and *BRAF* mutations in clinical glioma samples. We further developed a numeric expression for an objective mutation indicator, the HRM-mutation index (MI).

## Materials and Methods

### DNA preparation

Brain tumor samples were obtained from patients during surgery at Kyushu University Hospital. A section of tumor tissue was snap-frozen in liquid nitrogen and stored at -80°C. Tumor DNA was isolated from the frozen blocks, using a QIAamp DNA Mini Kit (Qiagen, Tokyo, Japan). Histological diagnoses were made by senior neuropathologists according to World Health Organization guidelines. For the initial *IDH1/2* analysis, surgically obtained tissue samples were collected from 192 patients, including 91 glioblastomas, 5 gliosarcomas, 23 anaplastic astrocytomas, 29 anaplastic oligodendrogliomas, 5 anaplastic oligoastrocytomas, 20 diffuse astrocytomas, 12 oligodendrogliomas, 1 oligoastrocytoma, 1 gliomatosis cerebri, 1 astroblastoma, 1 anaplastic pilocytic astrocytoma, 1 pleomorphic xanthoastrocytoma, 1 dysembryoplastic neuroepithelial tumor, and 1 histologically normal tissue sample. As a validation set for the *IDH1* analysis, we collected 46 additional glioma tissue samples whose corresponding pathological specimen for IHC analysis were available, including 31 glioblastomas, 2 anaplastic astrocytomas, 12 diffuse astrocytomas, and 1 oligodendroglioma. Tumor tissue samples collected from 52 patients were used for the *BRAF*^*V600E*^ mutation analysis; these included 24 pilocytic astrocytomas, 4 pleomorphic xanthoastrocytomas, 6 gangliogliomas, 14 adamantinomatous craniopharyngiomas, and 4 papillary craniopharyngiomas. Tumor DNA was also isolated from formalin-fixed paraffin-embedded (FFPE) specimens, using a RecoverAll^™^ Total Nucleic Acid Isolation Kit for FFPE (Thermo Fisher Scientific KK, Yokohama, Japan). Corresponding nontumor DNAs were isolated from a blood sample from the same patient using the QIAamp DNA Blood Mini Kit (Qiagen). The present investigation was approved by the Ethics Committee of Kyushu University and complied with the current laws of the country in which it was performed. Written informed consent was obtained from all participants.

### Primers

**Fig 1:**
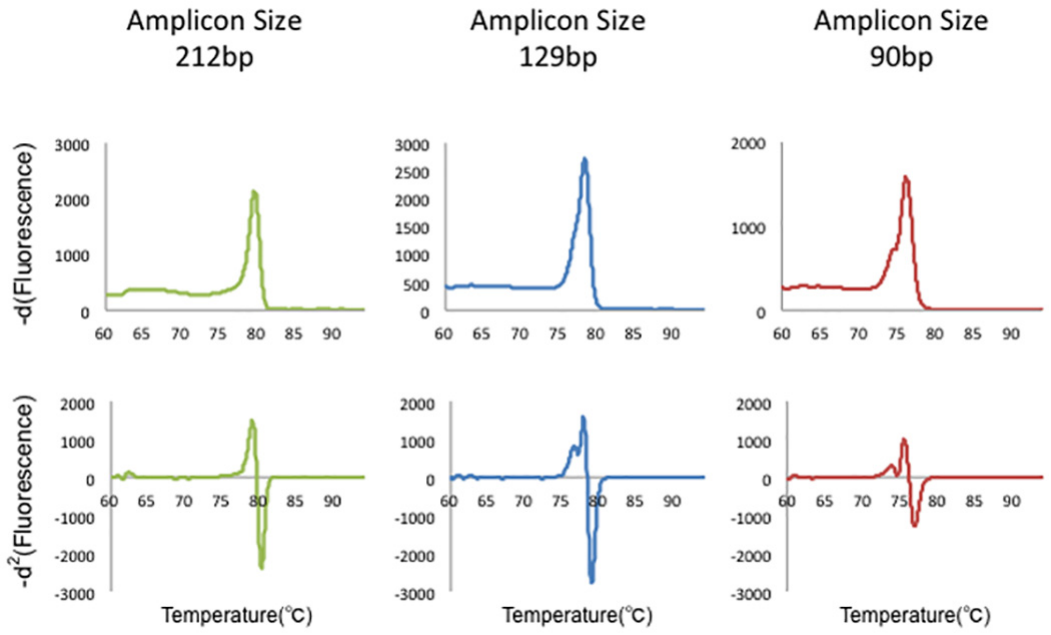
HRM analysis using different amplicon lengths. Multiple amplicon lengths (90, 129, and 212 bp) were tested for mutation analyses in order to optimize the HRM analysis. First row: -*d*^1^ curve; second row: -*d*^2^ curve. The 90-bp amplicon showed the most interpretable heteroduplex-derived peaks.

Primers for the amplification of genomic DNA were designed using *Primer3Plus* (http://www.bioinformatics.nl/cgi-bin/primer3plus/primer3plus.cgi/). In silico PCR applications were used to verify the theoretical specificity of the primers (http://genome.ucsc.edu/cgi-bin/hgPcr). Amplicon size was optimized experimentally (Fig 1), and, according to the results of this analysis, subsequent studies were performed using amplicons of 90–110 bp (see Table 1 for primer sequences).

**Table 1:** Primer sequences for high resolution melting analysis.

Gene	Primer sequence	Primer Tm (°C)	Amplicon size
*IDH1*	F: 5′-GGCTTGTGAGTGGATGGGTA-3′	60.9	90 bp
	R: 5′-GCAAAATCACATTATTGCCAAC-3′	59.4	
*IDH2*	F: 5′-AGCCCATCATCTGCAAAAAC-3′	60.1	102 bp
	R: 5′-CTCCACCCTGGCCTACCT-3′	60.1	
*BRAF*^*V600E*^	F: 5′-GAAGACCTCACAGTAAAAATAGGTGA-3′	59.2	102 bp
	R: 5′-CCACAAAATGGATCCAGACA-3′	59.3	

F: forward, R: reverse

### High-resolution melting

Whole HRM reactions were performed with 16.6 ng of DNA, 7.47 pmol/L each primer, and 10 μL of MeltDoctor HRM Master Mix (Applied Biosystems, Tokyo, Japan) in a total volume of 20 μL, according to the manufacturer’s protocol. A 7500 Fast Real-Time PCR System (Applied Biosystems) was used for amplification, and the cycling conditions were as follows: an initial denaturation of 10 min at 95°C; 40 cycles of 95°C for 15 s, 60°C for 1 min; and a dissociation cycle consisting of 95°C for 15 s, 60°C for 1 min, and 95°C for 15 s. Auto-call genotyping data determined by differentiation of the normalized and temperature-adjusted melting curves for analyzed samples were acquired using the HRM v2.0 software (Applied Biosystems).

### Sanger sequencing

PCR products were purified using ExoSap-IT (Affymetrix/USB, Santa Clara, CA, USA). Thereafter, cycle sequencing was carried out using BigDye^®^ Terminator v1.1 (for *IDH1/2*) or v3.1 (for *BRAF*) Cycle Sequencing Kits (Applied Biosystems). Following purification, the electrophoresis and analyses were conducted using a PRISM^®^ 3100 Genetic Analyzer (Applied Biosystems). Raw data were analyzed using the phred/phrap/consed package (http://www.phrap.org/consed/consed.html#howToGet) for sequence determination.

### Immunohistochemistry

IHC for IDH1^R132H^ was performed on 6-μm-thick, formalin-fixed, paraffin-embedded tumor sections using an antibody specific for the mutant IDH1^R132H^ protein (H09, dilution 1:10; Dianova, Hamburg, Germany). The tissue sections were deparaffinized and dehydrated, followed by endogenous peroxide quenching in 3% H_2_O_2_ for 30 min. Heat-induced epitope retrieval was performed at 120°C for 10 min in citrate buffer. The sections were then incubated with blocking serum followed by incubation with mutant IDH1^R132H^ protein (H09, dilution 1:10; Dianova) overnight at 4°C. The staining procedure was performed using a Vectastain Elite ABC Kit (Vector Laboratories, Burlingame, CA, USA) and DAB according to the manufacturer’s instructions.

## Results

### Detection of IDH1 mutations by conventional HRM analysis

**Fig 2:**
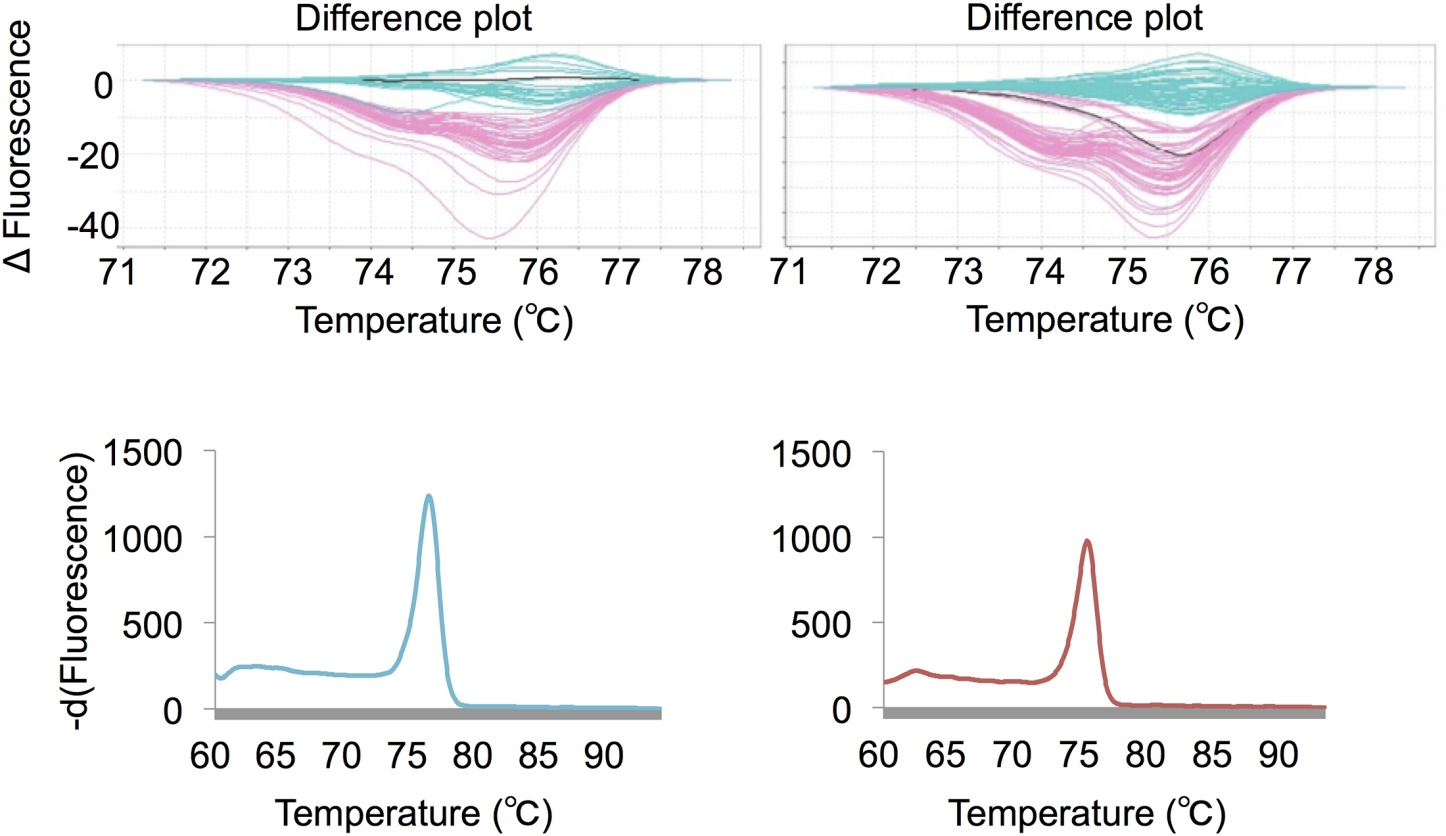
Discrepant results between a duplicate HRM analyses. Difference plots of discriminated wild-type calls and variant (i.e., mutant) calls from the first (left upper) and second (right upper) 96 runs, which are displayed as light blue and light red curves, respectively. A representative discrepancy for a duplicate HRM analysis, i.e., one with a wild-type call in the first run and a variant call in the second run, is shown as a black curve in both difference plots. Negative derivative curves of this discrepancy were similar to those of less interpretable plots (left lower, first run; right lower, second run).

We first tested the robustness of the conventional HRM analysis for the detection of mutations within glioma tissue samples. PCR and subsequent SS were performed on 192 tumor DNA samples for *IDH1* codon R132, and were classified as either seq-mut (n = 79; 77 R132H and 2 R132G) or seq-wt (n = 113). Duplicate HRM reactions (192 × 2; 384) for the corresponding DNA samples were performed, and the mutation status of each sample was interpreted independently. The concordance rate of SS and HRM was 94.5% (363/384), and sporadic discrepancies between duplicate HRM reactions were observed (21/192; 10.9%). We surveyed the difference plots and derivative curves of discordant results and speculated that the reproducibility of the analysis using HRM software is limited when the change in the melting curve shape is equivocal (Fig 2).

### Differential calculus analysis of the derivative melting curve

**Fig 3:**
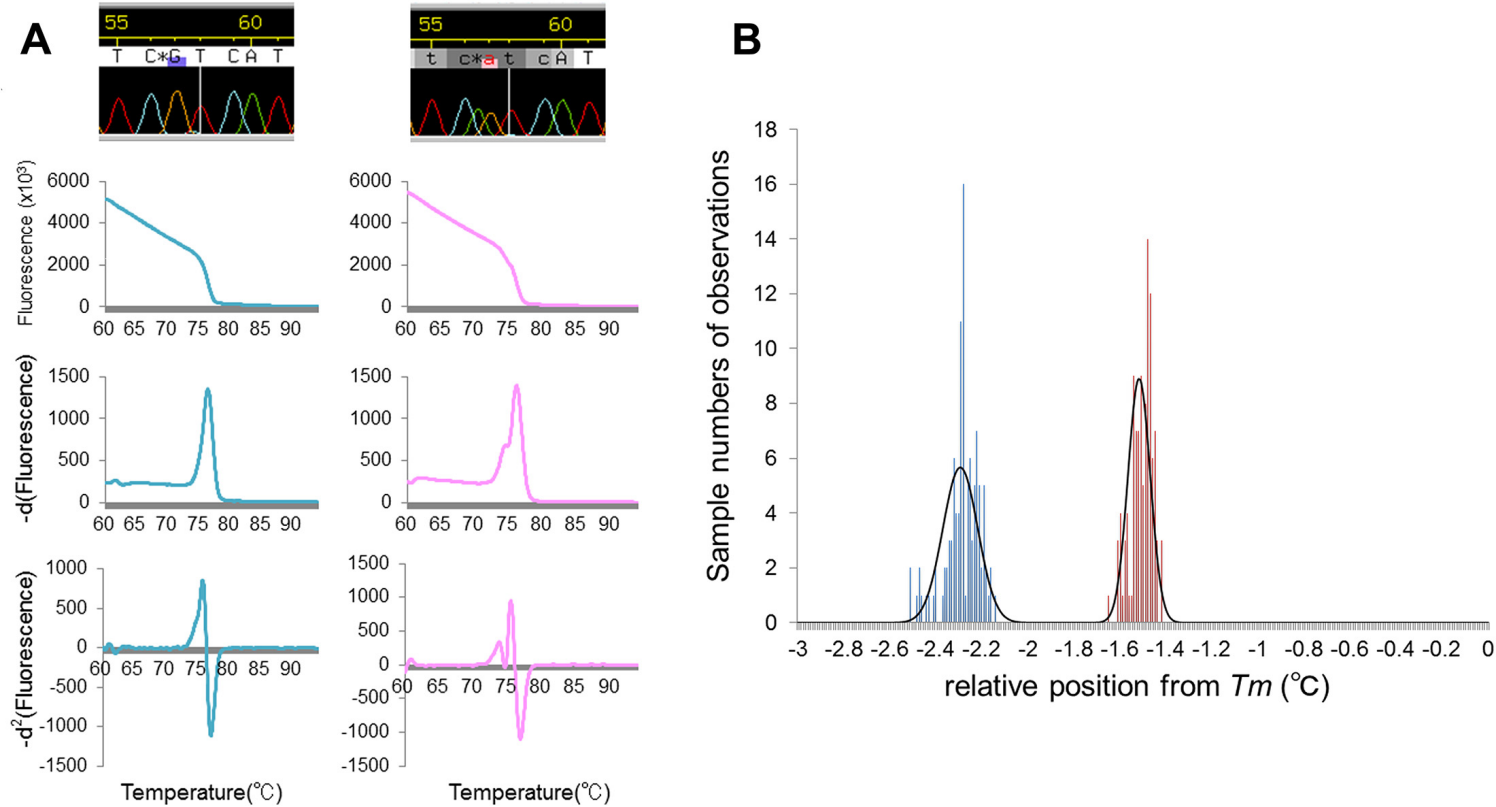
Differential calculus analysis of HRM data. A) Representative results of differential calculus analyses of HRM for *IDH1*^*R132*^. First row: Sanger sequencing; second row: fluorescence intensity curve; third row: -*d*^1^ curve; fourth row: -*d*^2^ curve. First column: A result of sequence-wild type DNA containing no heteroduplex-derived peaks in either the -*d*^1^ or -*d*^2^ curves. Second column: A result of sequence-mutant DNA. Whereas the heteroduplex-derived peak is recognized in the -*d*^1^ curve as a slight change of shape, the -*d*^2^ curve demonstrates a more distinct peak formation. B) Distributions of run-to-run variability in the low-temperature melting transition (LTMT) (blue) and high-temperature melting transition (HTMT) (red) positions from *Tm*. Bars indicate the number of observations within the bins of width 0.01°C. The curved black line shows the approximate normal distributions of LTMT and HTMT, in which the standard deviations were 0.076°C and 0.048°C, respectively.

**Fig 4:**
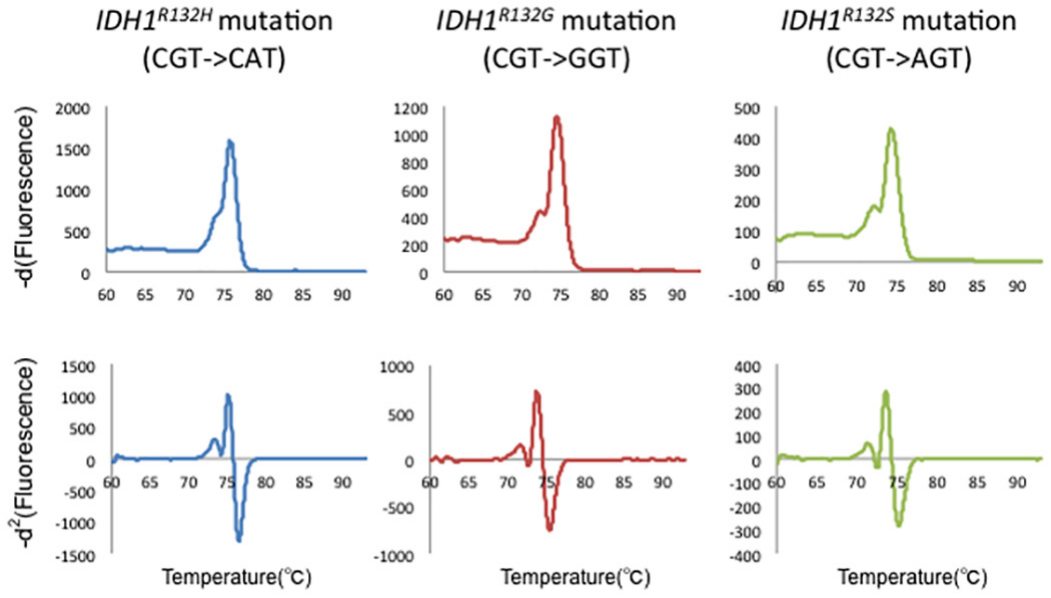
HRM analysis for rare IDH1 mutation genotypes. First row: -*d*^1^ curve; second row: -*d*^2^ curve. First column: *IDH1*^*R132H*^ genotype results; second column: *IDH1*^*R132G*^ genotype results; third column: *IDH1*^*R132S*^ genotype results. The tolerance to rare genotype variants is revealed.

Based on the hypothesis that the reliability of HRM depends on the precise discrimination ability of the melting curve, we developed a differential calculus approach in order to establish a robust analytical method. Negative first derivative values of fluorescence intensities, which are automatically provided in increments of 0.04 degrees by the default HRM software, were outputted. Using these values, the forward differences in increments of 0.04 degrees were then calculated. We edited approximated the negative second derivative curves (*-d*^2^ curve) by plotting these calculated values throughout the melting phase. The -*d*^2^ curve of the mutant DNA sample contained an easily interpretable extra peak derived from the heteroduplex, in marked contrast to that of the wild-type DNA sample, which showed a simple curve. Thus, this analysis enabled an unequivocal visual discrimination of mutation status (Fig 3A). The tolerance of our method to genotype variation was also revealed (Fig 4). To estimate the reproducibility of -*d*^2^ curve formations, triplicate HRM reactions from the same mutant DNA samples were performed in 36 variable well positions. Each *Tm* was outputted from the HRM software for 108 (36 × 3) experiments. The temperatures of the high melting transition and low melting transition within the extra heteroduplex peak in the -*d*^2^ curve were also recorded for each sample (S1 File). We defined the positions of the low-temperature melting transition (LTMT) and high-temperature melting transition (HTMT) relative to *Tm* as indicators of the reproducibility of extra peak formation because fluorescence magnitudes are essentially not reproducible owing to variation in starting templates and capillary optics between samples and well positions ^13)^. As shown in Fig 3B, the relative positions of LTMT and HTMT were fitted to Gaussian distributions with low standard deviations (0.076°C and 0.048°C, respectively) using the Shapiro-Wilk W test implemented in JMP software (Version 11.0.0; SAS Institute Inc.; Cary, NC, USA), which supports the reproducibility of extra peak formation in the -*d*^2^ curve.

Our approach improved the discrimination ability for mutation HRM curves; nonetheless, inter-observer variability cannot be completely excluded as long as the determination is dependent upon visual inspection or a comparison to wild-type curves. To establish an objective indicator of mutation status, we aimed to develop a numerical expression to identify the heteroduplex-derived peak. First, we defined the negative second derivative value of *t* (°C) as −*d*^2^*f*(*t*) We hypothesized that the difference in −*d*^2^*f*(*t*) between LTMT and HTMT would provide a numerical estimation of the mutation status. Accordingly, we defined the LTMT and HTMT zones within each 4*SD* range, which was calculated by the Shapiro-Wilk W test mentioned above, as *Z*_LTMT_ and *Z*_HTMT_, respectively.

Hence,

$$Z_{LTMT}=\{t;\;Tm-Ave_{LTMT}-4SD_{LTMT}<t<Tm-Ave_{LTMT}+4SD_{LTMT}\}.$$

$$Z_{HTMT}=\{t;\;Tm-Ave_{HTMT}-4SD_{HTMT}<t<Tm-Ave_{HTMT}+4SD_{HTMT}\}$$

Subsequently, we subtracted the infimum −*d*^2^*f*(*t*) of *Z*_HTMT_ from the supremum −*d*^2^*f*(*t*) of *Z*_LTMT_ and defined the resulting value as the HRM-MI as follows:

$$HRM-MI=\underset{t\in Z_{LTMT}}{\text{sup}}\left(-d^{2}f\left(t\right)\right)-\underset{t\in Z_{HTMT}}{\text{inf}}\left(-d^{2}f\left(t\right)\right)$$

(Fig 5A and 5B).

**Fig 5:**
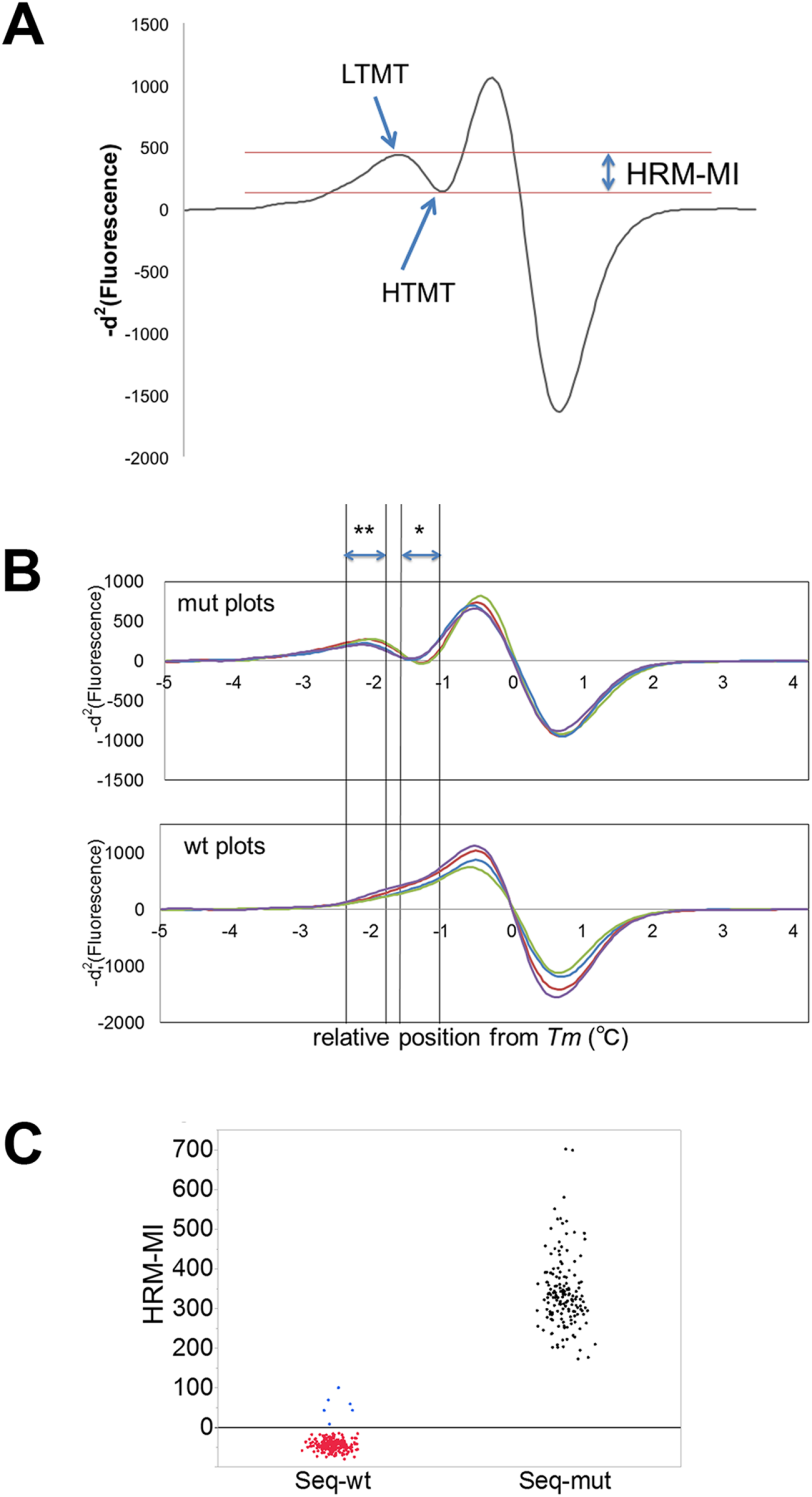
HRM-MI assay. A) Representation of the HRM-Mutation Index (HRM-MI), which is defined as the difference between the low-temperature melting transition and high-temperature melting transition. B) Representative -*d*^2^ curves of mutated and wild-type DNA samples. The *x*-axis indicates the position relative to *Tm*. A single asterisk shows *Z*_HTMT_, and double asterisk shows *Z*_LTMT_. Mutated DNA samples presented positive values of HRM-MIs owing to heteroduplex-derived peaks (upper graph). Conversely, wild-type DNA samples showed simple ascending curves between *Z*_HTMT_ and *Z*_LTMT_, and negative HRM-MI values (lower graph). The colors represent different samples. C) Distribution plots of HRM-MI for 192 DNA samples analyzed for *IDH1*^*R132*^. All-HRM-MI values of sequence-mutant (seq-mut) DNA samples were distributed in the positive range (black plots). The corresponding values for all wild-type (seq-wt) DNA samples were negatively dispersed (red plots), except for six values (blue plots) obtained from the duplicated results of three DNA samples.

**Fig 6:**
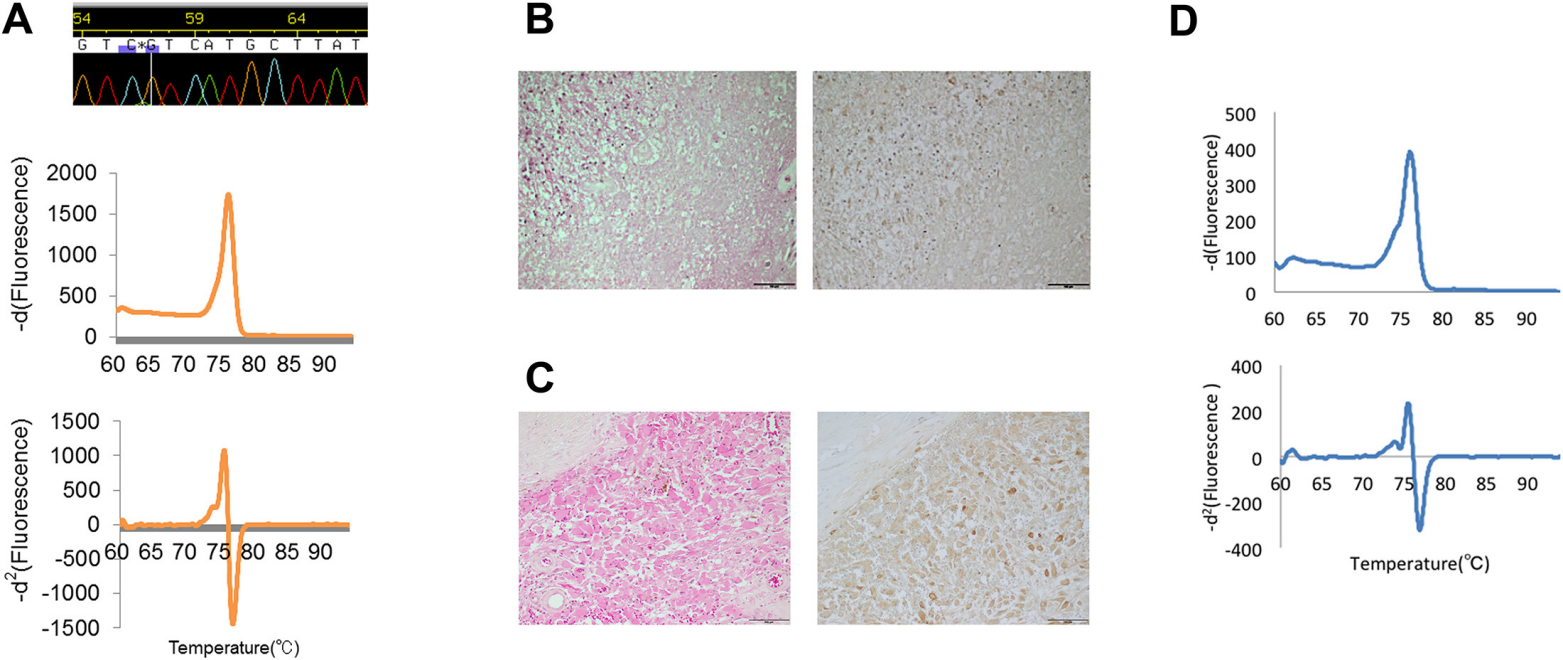
Analysis of IDH1 mutation in a damaged tumor tissue sample. A) An example of genetic and pathological findings of a tumor tissue specimen showing discrepant results between SS and HRM. SS called the genotype as the wild type, and the negative derivative curve presented an equivocal change in shape; however, the heteroduplex derived peak appeared in the *-d*^2^ curve, indicating the presence of a mutation (A). Pathological findings revealed that the corresponding tumor specimen consists of heterogeneous necrotic tissue (B, left: H&E; right: IHC of anti-IDH1 R132H) and focal proliferation of IDH1 R132H-positive tumor cells (C, left: H&E; right: IHC of anti-IDH1 R132H) D) Analysis result of the DNA extracted from FFPE specimen. The heteroduplex-derived peak is recognized in both the -*d*^1^ curve and the -*d*^2^ curve.

**Fig 7:**
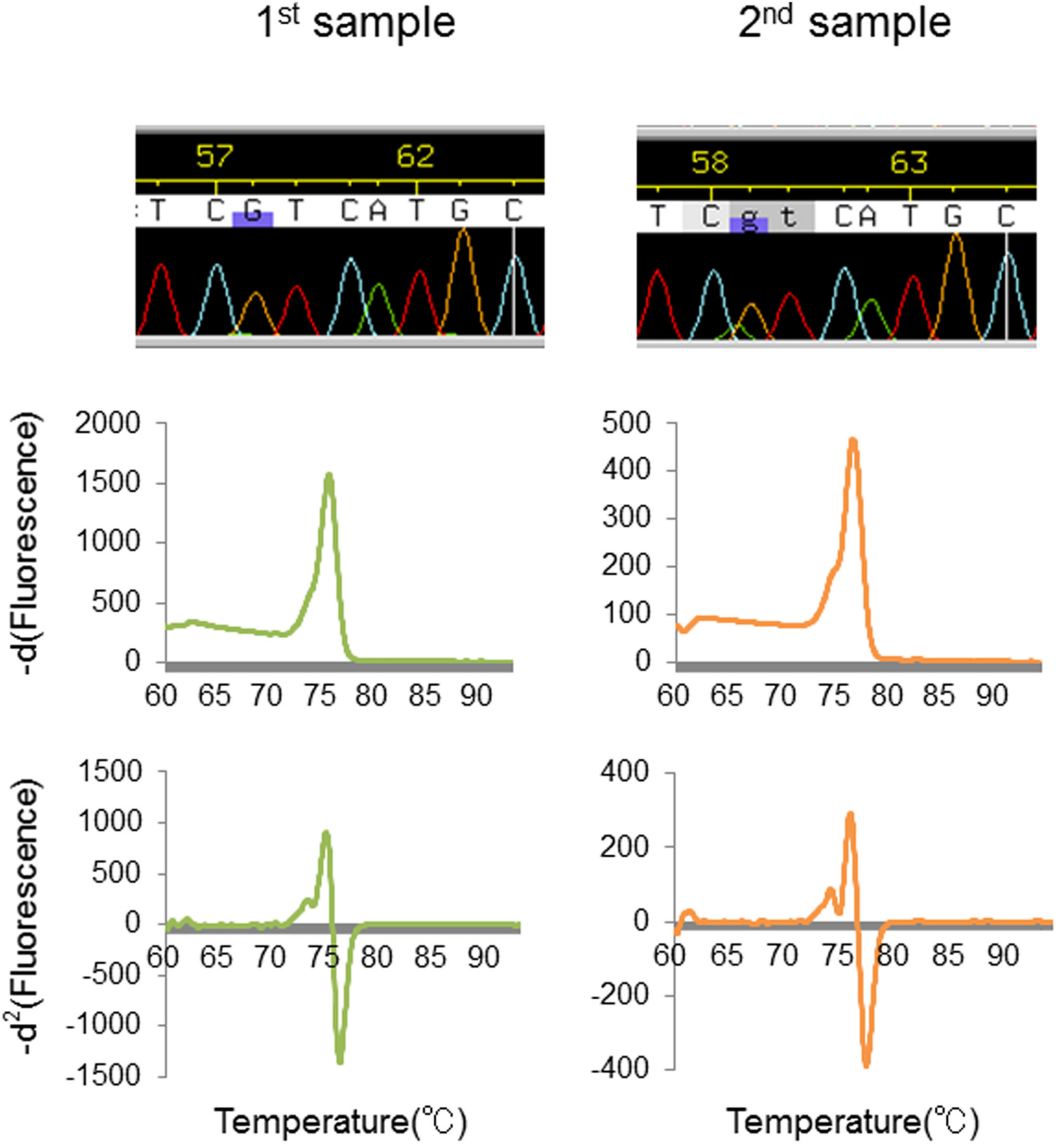
Analysis of IDH1 mutation in an inappropriate tissue sample. Analysis of a tumor tissue showing a sampling discrepancy. Whereas wild-type calls were obtained by SS and conventional HRM for the first sampling, the mutation was detected using our present method based on a heteroduplex-derived curve that presented a positive HRM-MI value (first column). The second sampling confirmed the mutation, with consistent results among the three approaches (second column).

We analyzed the duplicate HRM results of the 79 seq-mut DNA samples and calculated the HRM-MIs. As a control, the corresponding values were calculated for -*d*^2^ curve data of 113 seq-wt DNA samples. All of the HRM-MI values of the seq-mut DNA samples were distributed in the positive range (Fig 5C and S2 File). In contrast, HRM-MI values for the seq-wt DNA samples were negatively distributed, except for six values, which were derived from duplicated analyses of three identical DNA samples (Fig 5C). Among them was a tumor tissue sample obtained by surgery for the regrowth of an anaplastic astrocytoma after radiation treatment. Immunohistochemistry with the IDH1^R132H^ antibody using the corresponding tumor specimen revealed highly necrotic tissue containing sparsely distributed mutant IDH1-positive tumor cells (Fig 6A–6C). We analyzed an additional DNA sample extracted from the tumor-rich part of the tissue, using a formalin-fixed, paraffin-embedded specimen, and clear mutation results were obtained by HRM (Fig 6D). Thus, we were able to precisely detect *IDH1* mutations, even in damaged tissue, which could have been missed by SS. The remaining two samples, which showed discrepant results between HRM-MI and SS, were obtained from the surgeries of two oligodendrogliomas. In both cases, we performed HRM and SS analyses using DNA samples extracted from another part of the tumor and obtained clear mutational results (Fig 7), indicating that our newly developed analysis is precise, even for inappropriate tissue samples. In summary, HRM-MI enabled the unequivocal determination of mutations and, as shown in Table 2, provided improved reproducibility and concordance rate with SS results, significantly higher than conventional HRM (p < 0.01, Fisher's exact test).

**Table 2:** Concordance rates between different analytical methods.

Target Gene	Concordance rates between duplicates using conventional HRM	Concordance rates between duplicates using new method	Concordance rate of SS and conventional HRM	Concordance rate of SS and new method
*IDH1*	89.1% (171/192)	100%	94.5%(363/384)	98.4% (378/384)
*BRAF*	90.4% (47/52)	100%	95.2% (99/104)	100% (104/104)

To validate the reliability of our new analytical method, we analyzed *IDH1* mutations, using a second sample set by HRM, SS, and IHC. Discrepant results between SS and IHC were only observed for one sample (97.8% concordance; 45/46), which had the rare *IDH1*^*R132S*^ genotype. While discrepancies between the results of conventional HRM and SS were seen as frequently as those in the initial sample set (93.5% concordance; 43/46), the results of HRM-MI and SS were completely concordant, validating the high reliability of our new method.

**Fig 8:**
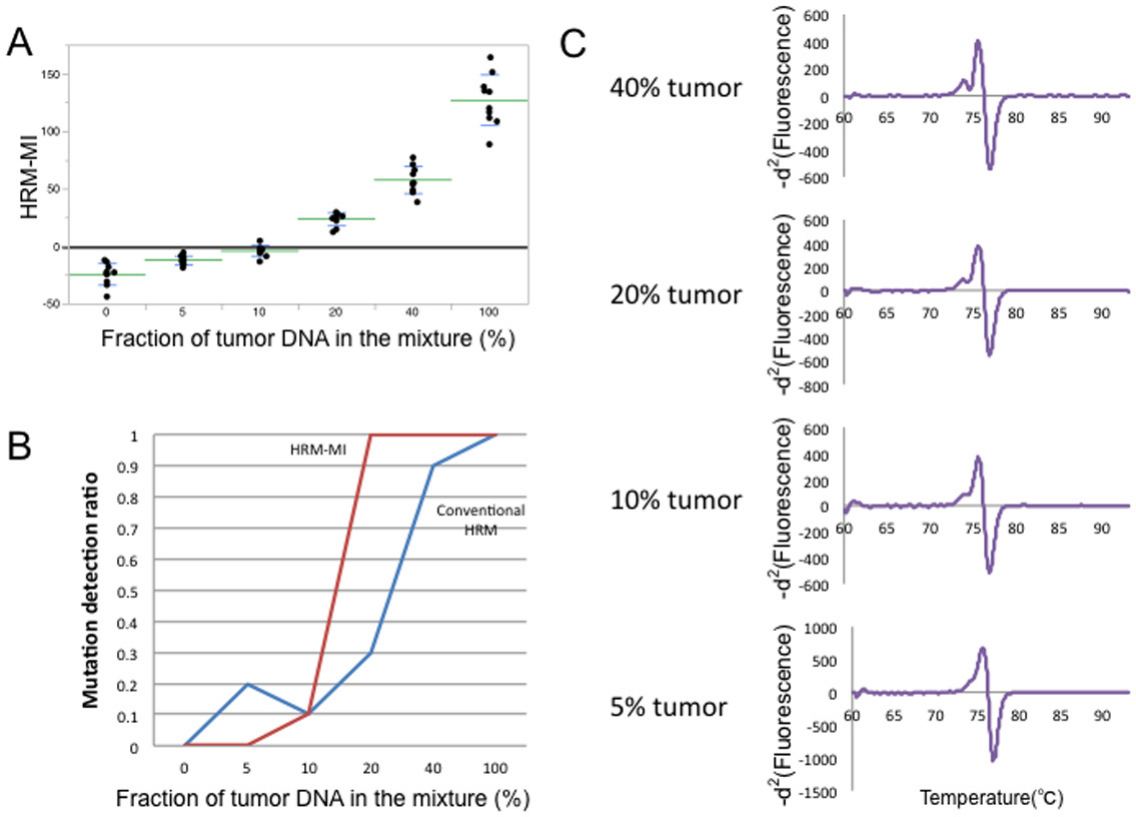
HRM analysis using mixed samples. A) Distribution plots of HRM-MI results obtained using an HRM analysis with mixed samples. The *x*-axis shows the fraction of tumor DNA in the mixture. The *y*-axis shows the HRM-MI value. Each dot represents the HRM-MI value for each assay, performed with multiple replicate across the 6 different mixing ratios. Green and blue lines indicate mean and SD HRM-MI values, respectively. B) Mutation detection ratios for HRM-MI (red line) and conventional HRM analyses (blue line). At a mixing ratio of 20% (i.e., 20% tumor DNA and 80% nontumor DNA), the sensitivities of the conventional HRM method and HRM-MI were 30% and 100%, respectively. C) The representative -*d*^2^ curves of samples with tumor-to-nontumor DNA ratios. Even the 10% tumor DNA sample presented an interpretable heteroduplex-derived peak.

To further estimate the sensitivity of our method, we performed reconstitution experiments in which a pair of DNA samples from a tumor and healthy tissue (blood) of the same individual were mixed at various ratios and subjected to HRM analyses (Fig 8 and S3 File)). Using 20% tumor DNA and 80% nontumor DNA, the sensitivities of the conventional HRM method and HRM-MI were 30% and 100%, respectively. Additionally, a visual inspection of mutations was still possible using our method, even using samples with 10% tumor DNA and 90% nontumor DNA. These results validate the high sensitivity of our method, which can tolerate heterogeneous DNA samples that cannot be evaluated using conventional HRM analysis.

### IDH2 and BRAF^V600E^ mutation analysis

**Fig 9:**
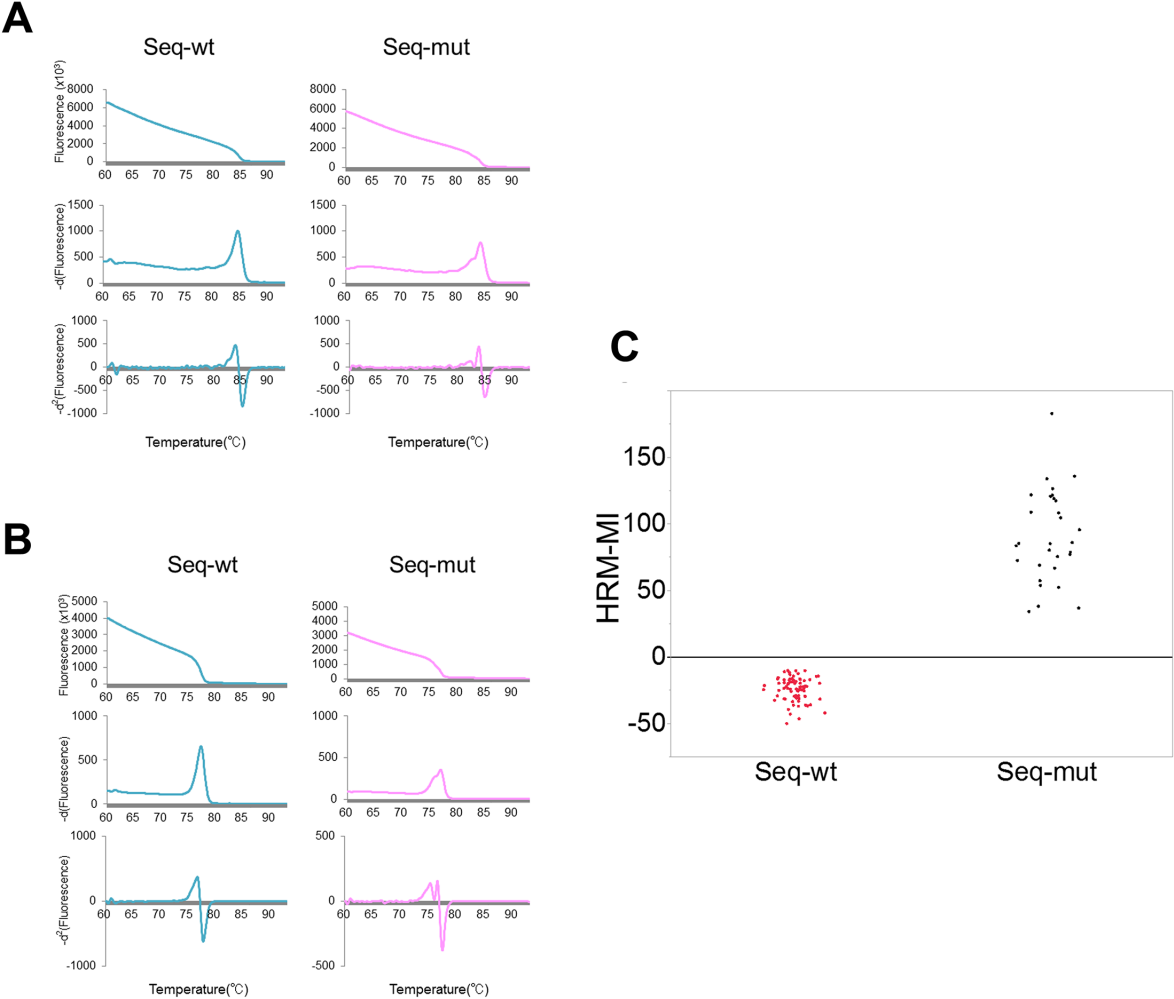
HRM analysis for hotspot mutations of IDH2 and BRAF. First column: Sequence-wild-type DNA results, including no heteroduplex-derived peak in either derivative curve. Second column: Clear heteroduplex-derived peaks are seen in both derivative curves of the sequence-mutant DNA. C) Distribution plots of HRM-MI for 52 DNA samples analyzed for *BRAF*^*V600E*^. HRM-MI values completely match the sequence results.

To test the universality of our method, we analyzed additional mutations. *IDH2*^*R172*^ mutations were analyzed for the 192 glioma samples by HRM and SS, and mutation status was interpreted independently. *IDH2* mutations were detected in only 4 samples by SS, so a statistical analysis was not performed; however, the -*d*^2^ curves of mutational DNA samples presented demarcated heteroduplex peaks, which can be precisely interpreted by visual inspection (Fig 9A). *BRAF*^*V600E*^ mutations were analyzed using the second sample set, including gliomas and other brain tumors with a high prevalence of this mutation, and the same assay was performed. Using SS, 15 mutants and 37 wild-type samples were observed. The mutant DNA samples showed marked heteroduplex peaks in the -*d*^2^ curves, and the reproducibility of the position of the mutation was also confirmed (Fig 9B). HRM-MI scores further revealed a clear discrimination between the mutant and wild-type samples (Fig 9C and S4 File). The mutation results of HRM-MI were completely concordant with those of SS (Table 2). These results not only validated our approach, but also indicated that our method is applicable to the detection of mutations in tumors other than gliomas.

## Discussion

Various platforms, such as IHC, SS, pyrosequencing (PS), and HRM, are available to analyze genetic mutation profiles in clinical tissue samples. Among them, IHC is the most popular approach and can be performed using clinical pathology techniques and specimens. Its low cost, lack of a DNA purification step, and high resolution (i.e., its ability to detect even single mutant cells) support the potential of IHC for clinical applications; however, IHC can detect only a targeted mutation, and inter-laboratory and inter-observer variabilities are not negligible [19]. SS is the gold-standard technique to confirm mutations. However, this technique requires complex laboratory procedures and has a modest limit of detection [18]. PS and HRM are alternative platforms to detect mutations in tumor tissue samples [20–21]. PS is a bioluminescence technique that can identify mutations at predetermined positions with higher sensitivity and less labor than SS; however, the cost of reagents is relatively high in comparison to SS, and the assay must be performed on a special instrument, i.e., a pyrosequencer [18].

DNA melt curve profiles generated by HRM distinguish small differences between sequences, which enables reliable mutation scanning by most quantitative PCR machines [22]. HRM is a simple and cost-effective platform with a low risk of contamination, as this technique follows a simple protocol using closed-tube reactions [23]. Moreover, HRM has previously been shown to be sufficiently sensitive to detect mutations in highly heterogeneous samples [15]. These advantages could facilitate the application of HRM to clinical diagnostics. Recent studies have proposed a modified HRM in combination with rapid DNA extraction and PCR to enable the intraoperative detection of mutations for surgical glioma specimens [24–26]. HRM mutation scanning is conventionally acquired using pre-equipped software. In this software, shape changes are detected between the normalized and overlaid melting curves displayed on difference plots [27]. In this mutation-scanning mode, HRM has a tendency to identify wild-type DNA samples as “variant” [28]. More than other platforms, HRM yields equivocal results, especially for heterogeneous clinical samples [18]. Heterogeneity in tumor tissue is inevitable because ancillary cells are essential for tumor maintenance and tumor cells do not have uniform genetic changes in clinical tumor tissue samples [29]. In the present study, we proposed a novel analytical method using HRM that enables unequivocal detection of mutations and a numerical indicator of mutation status by which inter-observer discrepancies can be excluded. HRM combined with co-amplification at lower denaturation temperature-PCR is another approach for mutation detection in highly heterogeneous clinical samples, but it requires a customized PCR program [30]. The high objectivity and universality achieved by our analytical method might promote the application of HRM to clinical diagnostics. The primary limitation of our method at present is the inability to directly apply it to various quantitative PCR platforms. Recommended settings and equipped software vary among instruments [31]; consequently, instrument-to-instrument discrepancies cannot be ignored. Accordingly, it is necessary to perform a pilot experiment using mutant DNAs to optimize the temperature zones for the detection of HRM-MI for each instrument. However, our analytical method is based on a simple mathematical approach using raw data for dissociation profiles, and we anticipate a satisfactory degree of universality among platforms. Another limitation of our method is the potential for interference by variant sequences. Our method tolerated sequence variation, as shown in Fig 4, based on samples with the rare genotypes *IDH1*^*R132G*^ and *IDH1*^*R132S*^; however, other mutant genotypes or variant sequences, such as SNPs, were not evaluated in the present study. The hotspot mutations detected in neoplasms, such as *IDH* or *BRAF* mutations, are located at specific genetic loci leading to gains-of-function [5]. These specific loci are within highly preserved, usually coding regions, where variant sequences are expected to be very rare. Nonetheless, it is necessary to check for variant sequences when designing amplicons for mutation analyses using our method.

*IDH* mutations produce 2-hydroxyglutarate and are related to gliomagenesis by inducing a hypermethylator phenotype [32–35]. A previous study revealed impairment of glioma cell growth by a mutant *IDH1* inhibitor, which is anticipated to be utilized for future molecular-targeted therapy [36]. Within *BRAF*, p.V600E is the most frequent mutation; it constitutively activates RTK signaling pathways and is prevalent in various cancers [37]. *BRAF*-targeted therapy has already been shown clinical efficacy for metastatic melanoma [38]. Moreover, recent studies have demonstrated the antitumor activity of a *BRAF* inhibitor in preclinical models of *BRAF*^*V600E*^-mutant tumors, including gliomas [39]. Simple analytical methods with broad clinical applications for the detection of mutations will be essential for the generalization of molecular-targeted therapies. The method developed in this study improves the reliability and objectivity of mutation analyses using HRM and accordingly have applications to molecular-targeted therapies, especially for heterogeneous tumors, such as gliomas.

## Supporting Information

S1 File The raw data of triplicate HRM reactions from the same mutant DNA samples. Triplicate HRM reactions from the same mutant DNA samples were performed in 36 variable well positions. Each *Tm* was outputted from the HRM software for 108 (36 × 3) experiments. The temperatures of the high melting transition and low melting transition within the extra heteroduplex peak in the -*d*^2^ curve were also recorded for each sample.
(XLSX)

S2 File The raw data for the calculation of HRM-MIs using *IDH1* mutant and wildtype samples. The 384 HRM-MIs were calculated by the duplicate HRM results of the 79 *IDH1*-mutant and 113 wildtype DNA samples.
(XLSX)

S3 File The raw data of HRM analysis using mixed samples. Decuplicate HRM reactions from mixed DNA samples were performed. Each *Tm* was outputted from the HRM software and the temperatures of the high melting transition and low melting transition were recorded for the calculation of HRM-MIs.
(XLSX)

S4 File The raw data for the calculation of HRM-MIs using *BRAF*^*V600E*^ mutant and wildtype samples. The 104 HRM-MIs were calculated by the duplicate HRM results of the 15 BRAF-mutant and 37 wildtype DNA samples.
(XLSX)

## Abbreviations

HRM: high resolution melting
SS: Sanger sequencing
HRM-MI: HRM-Mutation-Index
IHC: immunohistochemistry
PCR: polymerase chain reaction
seq-mut: sequencing-mutant
seq-wt: sequencing-wild-type
-*d*^1^ curve: negative derivative curve
-*d*^2^ curve: negative second derivative curve
LTMT: low-temperature melting transition
HTMT: high-temperature melting transition
PS: pyrosequencing
